# Is hyperlipidemia a potential protective factor against intraoperative awareness in cardiac surgery?

**DOI:** 10.1186/s13019-016-0454-7

**Published:** 2016-04-12

**Authors:** Qingshui Zheng, Qian Wang, Chaoqun Wu, Zhifa Wang, Hushan Ao

**Affiliations:** Department of Anesthesiology, Fuwai Hospital, Peking Union Medical College, Chinese Academy of Medical Sciences, Beijing, China; Department of Anesthesiology, Ordos Central Hospital, Inner Mongolia Medical University, Inner Mongolia, China; Fuwai Hospital, Peking Union Medical College, Chinese Academy of Medical Sciences, Beijing, China

**Keywords:** Anesthesia, Cardiac surgery, Hyperlipidemia, Intraoperative complications, Intraoperative awareness, Protective factor

## Abstract

**Background:**

Intraoperative awareness is a dreaded complication that leads to psychological sequelae such as posttraumatic stress disorder, especially in patients undergoing cardiac surgery. This study investigated the incidence of awareness among patients receiving cardiac surgery and sought to identify the risk factors contributing to intraoperative awareness.

**Methods:**

Patients with informed consent undergoing cardiac surgery from June to September in 2012 were enrolled. At least one structured interview was performed postoperatively with the modified Brice Interview Questionnaire to identify intraoperative awareness as confirmed awareness, possible awareness, and no awareness. Confirmed awareness events reported by patients were classified into different categories with the Michigan Awareness Classification Instrument. The questionnaire results were combined with the patient medical records. A logistic regression model was used to analyze the risk factors that may have led to intraoperative awareness.

**Results:**

An estimated 2136 patients were included, and 1874 patients completed at least one interview. 83 patients (4.4 %) were identified as possible or confirmed awareness, among which 46 (2.5 %) reported confirmed awareness. Patients who experienced confirmed awareness were mostly of Class 1 and 2, 15 and 24 patients respectively, which represented isolated auditory and tactile perceptions. And 11 patients reported feelings of distress intraoperatively. Hyperlipidemia was associated with intraoperative awareness (OR = 0.499, 95 % CI = 0.252–0.989, *p* = 0.043) and using chi-square test, however, no significance was found with logistic regression.

**Conclusion:**

Patients undergoing cardiac surgery are at high risk for intraoperative awareness. Distress is a common feeling in patients with intraoperative awareness. Hyperlipidemia is a potential protective factor for intraoperative awareness in cardiac surgery.

## Background

Intraoperative awareness is defined as “…the postoperative recollection of events occurring during general anesthesia”, [1] which is an infrequent but dreaded complication that occurs in patients undergoing general anesthesia at an incidence of 0.10–1.05 % among non-cardiac surgery reports (Table 1). It is relatively more frequent in cardiac surgery: reported as 6 % in China in 2009 by Xu et al. [2] and an unexpectedly high incidence of 23 % by Goldman et al. [3] in 1987. We found a rate of 3.0 % in 2013 [4]. Though Awareness with explicit recall is not a lethal complication, psychologically adverse symptoms, such as post-traumatic stress disorder (PTSD) characterized by ‘re-experiencing, avoidance, and physiological hyperarousal’ [5] may develop, commonly and persistently, in the patients who experience anesthesia awareness [6]. Identification of risk factors contributing to awareness deserves further study, and corresponding strategies to prevent awareness during general anesthesia are justified.

**Table 1:** Incidence of intraoperative awareness in reported studies

Author	Country	Year	Incidence (%)
NON-CARDIAC SURGERY			
Myles, et al. [9]	Australia	2000	0.10
Akavipat, et al. [10]	Thailand	2009	1.05
Sebel, et al. [11]	U.S.A.	2004	0.13
Sandin, et al. [12]	Sweden	2000	0.16
Xu, et al. [2]	China	2009	0.41
CARDIAC SURGERY			
Maunuksela [16]	–	1977	5.0
Goldmann, et al. [3]	–	1987	23
Gordon, et al. [15]	South Africa	1994	1.1
Wang Yun, et al. [17]	China	2005	6
Xu, et al. [2]	China	2009	6
Qian Wang, et al. [4]	China	2013	3.0

## Methods

### Patient population

The study was approved by the Institutional Review Board of Fuwai Hospital in Beijing, China. Patients with Written informed consents receiving selective cardiac surgery under general anesthesia from June to September in 2012 were enrolled. Inclusion criteria were patients older than 18 years, with normal mental status, and able to provide informed consent. Patients were excluded if they did not meet the criteria or were not able to complete the follow-up questionnaires: (1) died intra- or postoperatively in the hospital; (2) could not be extubated early within 3–6 days; (3) could not communicate readily; (4) had abnormal mental status.

A sample of 1525 patients was estimated initially based on our prior study [4] that found a rate of intraoperative awareness of 3.0 %. We took a possible loss-to-follow-up rate of 30 % into consideration and set the final sample target at 2136 patients.

### Conduct of the study

Individual practitioners, who were blinded to the study, made anesthetic algorithms, including anesthetic drugs and depth of anesthesia monitoring case-by-case. All patients were transferred to the Intensive Care Unit (ICU) for a period of postoperative sedation and ventilation, and then transferred to wards where the patients were awakened and extubated.

Each patient was interviewed by research staff with the modified Brice Interview [7]. The research staff classified each patient report into confirmed awareness, possible awareness, and no awareness on the basis of published definitions [8]. Events were classified according to the Michigan Awareness Classification Instrument [8]. Patients who reported awareness received follow-up interviews to determine if the events confirmed awareness. The occurrence of awareness during the ICU stay would be excluded.

### Statistical analysis

The patient medical records were retrieved and combined with postoperative questionnaire results. Descriptive statistics were used to describe the incidence of intraoperative awareness of cardiac surgery. Comparisons of continuous variables between the “Confirmed Awareness” and “No Awareness or Possible Awareness” groups were conducted with the independent sample *t*-test, and categorical variables between groups with Fisher’s exact test or chi-square test, with or without Yates’ continuity correction. *P* values of less than or equal to 0.05 were considered to indicate statistical significance. The statistical analyzes were performed with SPSS 21.0 (SPSS Inc., Chicago, IL).

## Results

### Patients

**Fig. 1:**
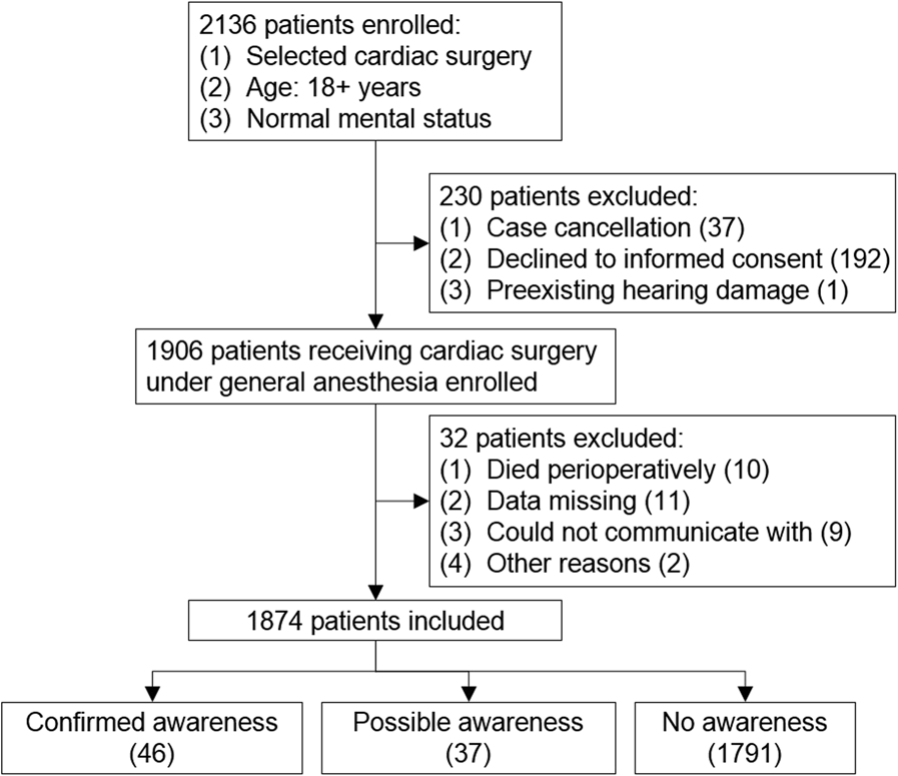
shows the screening and follow-up of patients undergoing cardiac surgery under general anesthesia

Figure 1 shows the selection of patients included. Of all the estimated 2136 patients, 230 were excluded because of rejection to or being not competent for informed consent, surgery cancelation, or preexisting hearing loss. One thousand nine hundred six patients entered the study during a 4-month period from June to September in 2012, and a total of 1874 patients completed the study. Thirty-two patients were excluded because they were lost to follow-up after surgery due to death (10 cases), insufficient or missing data (11 cases), being not competent for a structured interview, and other reasons (11 cases).

### Occurrence of intraoperative awareness

**Fig. 2:**
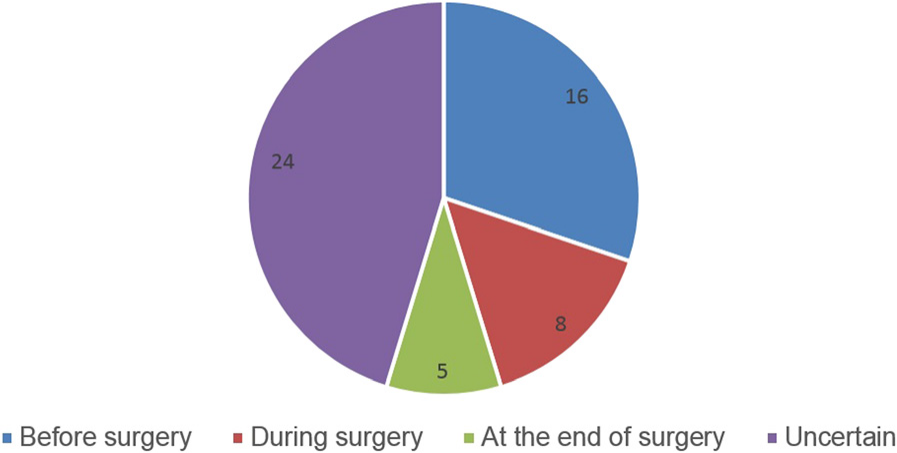
The timing of reported awareness. Patient who experienced intraoperative awareness reported at least one events. 16 events occurred before surgery; 8 events during surgery (the end of surgery not included); 5 events at the end of surgery; and 24 events were uncertain about the exact timing

Among the 1874 patients interviewed, 46 (2.5 %) were identified as confirmed awareness, 37 (1.9 %) as possible awareness, and 1791 (95.6 %) as no awareness. Detailed descriptions of recollections are listed in Table 2. Each patient who experienced confirmed awareness reported at least one event during general anesthesia. Figure 2 shows the timing of reported awareness events. Six-teen of the patients reported events that occurred during the period between anesthesia induction and skin incision, most of which were perceptions of intubation and central line placement; 18 reported events during surgery, not all known the exact timing of the surgery due to lack of exact markers of specific events; five reports were identified at the very end of the surgery; and 24 patients reported sounds, pains and perceptions but were not able to determine when they occurred.

**Table 2:** Descriptions of confirmed awareness reported by patients

Number	Gender and age	Surgery	Reported events	Michigan awareness classification
1	M/67	CABG	Formication; perception of central line placement; mild pain	Class 3
2	M/73	Bentall procedure	Precordial stabs; nausea	Class 2D
3	M/64	CABG	Operation on the chest	Class 2
4	M/30	Repair of aneurysm of aortic sinus, repair of VSD	Perception of central line placement	Class 2
5	F/53	MVR	Placement of nasal thermometer	Class 2
6	M/23	Modified Morrow procedure	A surgeon making explanations in a Tangshan accent	Class 1
7	M/65	Wheat procedure and CABG	Dreaming things back in decades ago; lower extremity operation	Class 2
8	M/66	CABG	Intubation	Class 2
9	M/40	Repair of aneurysm of aortic sinus	Intubation; operation, electroshock on heart	Class 2
10	M/67	CABG	Intubation, feeling nausea twice	Class 2D
11	M/60	CABG	Scalpel incision; voice from doctors	Class 2
12	M/60	CABG	Intubation; internal jugular vein catheterization	Class 2
13	M/65	CABG	Sensation of operation on chest and lower limb	Class 2
14	M/66	Resection of atrial myxoma, TVP	Feelings of bugs-like crawling on the chest	Class 2
15	M/38	ASD repair, TVP	Sensation of sounds from the respirator	Class 1
16	M/56	CABG	Heard the surgeons’ talking	Class 1
17	F/45	MVR, TVP	Heard the sounds of electric scalpel, like “chi, chi”	Class 1
18	F/18	ASD repair	Intubation; nausea; discomfort on the back	Class 2D
19	F/59	MVR, TVP	Operation	Class 2
20	M/49	CABG	Water dripping-like sounds of the machine; sensation of intubation and a severe feeling of nausea	Class 2D
21	F/56	CABG	Sensation of the operation; severe pain, being unable to move	Class 5
22	M/55	Correction of aberrant of pulmonary artery, TVP	Sounds of the machine in the operation room	Class 1
23	M/34	Replacement of aortic root and ascending aorta	Sounds of a scalpel and scissor	Class 1
24	F/51	Replacement of ascending aorta	Heard the surgeon talking about the electric defibrillations, of which only the third time worked	Class 1
25	F/60	MVR, AVR, TVP	Heard the surgeon said the operation was well finished; intubation; felt nausea twice	Class 2D
26	F/50	MVP	Dreaming, cannot afford details; heard the surgeon’s talking; central line placement	Class 2
27	M/58	CABG	Lower limb operation	Class 2
28	M/74	CABG	Intubation; felt nausea; sensation of the operation on the chest	Class 2D
29	M/62	CABG	Operation on chest with burning heat	Class 2
30	F/30	Repair of ASD, TVP	Sounds like sawing wood	Class 1
31	F/31	Repair of ASD, TVP	Voice, such as “pass the scalpel to me”	Class 1
32	M/43	Modified Morrow procedure	Operation on chest; severe pain, wanting for more anesthetics	Class 3D
33	M/60	CABG	Felt awake for a long time and felt like breathing with effort	Class 1D
34	M/52	Bentall procedure	Heard “chi-chi” like sounds	Class 1
35	M/46	MVR, AVR, TVP	Heard the surgeon said the operation will be finished in an hour; heard the “dong-dong” like sounds	Class 1
36	M/37	MVR	Dreaming of receiving cardiac surgery; sensed the central line placement	Class 2
37	F/48	MVR, TVP, and PDA repair	Scissors cutting on the chest; mild pain	Class 3
38	M/60	CABG	Operation on chest and lower limb	Class 2
39	M/41	CABG	Something dragged down from the chest; unable to move; central line placement	Class 4
40	M/59	MVR, CABG	“chi-chi” sound	Class 1
41	M/34	CABG	Dreaming of the lifetime in high school; heard the surgeon said the operation was almost finished	Class 1
42	F/58	CABG	Heard the surgeon said 5 bypass grafts were done, and the 2 nurses had done a great job	Class 1
43	M/40	AVR	Intubation; unable to move; afraid	Class 4D
44	M/57	CABG	Chest stuffy, unable to speak	Class 4D
45	M/56	Replacement of descending aorta	Intubation; nausea	Class 2D
46	M/54	CABG	Heard the surgeon said the operation was well done; felt his chest stabbed 4–5 times.	Class 2

*Abbreviations*: *CABG* coronary artery bypass grafting, *MVR* mitral valve replacement, *AVR* aortic valve replacement, *TVP* tricuspid valvuloplasty, *PDA* patent ductusarteriosus

**Fig. 3:**
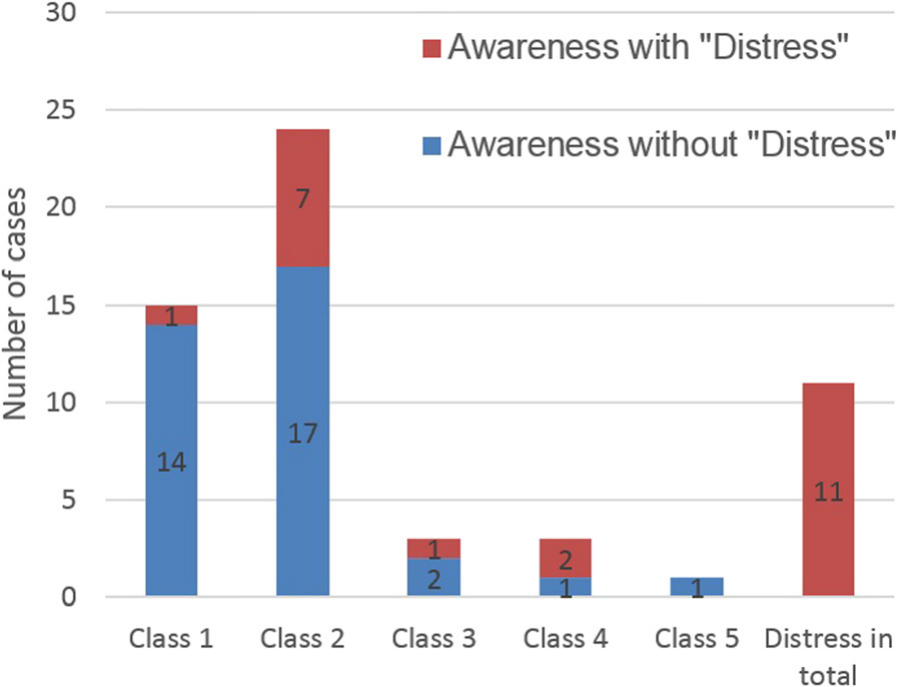
The Michigan Awareness Classification of Awareness. Class 1 denotes isolated auditory perceptions; Class 2 tactile perceptions (e.g., surgical manipulation or endotracheal tube); Class 3 pain; Class 4 paralysis (e.g., feeling one cannot move, speak or breathe); Class 5 paralysis and pain. “Distress” was assessed when a patient reported of fear, anxiety, suffocation, sense of doom, sense of impending death, etc. The red column indicates the patients with distress, and blue column indicates the patients without distress

Reports of confirmed awareness were classified into six levels, from Class 0 to 5, and an additional D for “distress” was included, according to the Michigan Awareness Classification Instrument (Fig. 3).

### Risk factors for intraoperative awareness

Table 3 shows the demographic characteristics of patients with confirmed awareness, compared with patients who had no awareness or possible awareness. Patients who reported confirmed awareness had a lower rate of hyperlipidemia (23.9 % vs. 38.6 %, *p* = 0.043, OR = 0.499, 95 % CI = 0.252–0.989) and had a shorter duration of surgery (195.0 ± 52.0 min vs. 225.2 ± 82.4 min, *p* = 0.013), compared with the other patients. And patients who were young than 60 years old age showed a higher rate of awareness than those who were elder than 60 (1.9 % vs. 0.5 %, *p* = 0.026). No differences were seen in other pre- and intraoperative factors. And no significance was found between hyperlipidemia and awareness when age, hyperlipidemia and duration of operation were entered into logistic regression. (*p* = 0.159, OR = 0.604, 95 % CI = 0.300–1.218) (Table 4).

**Table 3:** Demographic characteristics of study population (*n* = 1874)

Characteristic	No awareness or possible awareness	Confirmed awareness	*P* Value
Age-no. (%)	–	–	0.026^*^
< 60 year old	1137 (60.7)	36 (1.9)	–
> 60 year old	691 (36.9)	10 (0.5)	–
Male-no. (%)	1212 (66.3)	33 (73.9)	0.28
Height-cm	165.7 ± 8.7	166.1 ± 8.1	0.80
Weight-kg	68.0 ± 12.4	67.7 ± 12.9	0.88
BMI	24.8 ± 6.5	24.4 ± 3.7	0.67
Blood group-no. (%)	–	–	0.34
A	511 (28.1)	16 (34.8)	–
B	607 (33.4)	18 (39.1)	–
AB	188 (10.4)	2 (4.3)	–
O	510 (28.4)	10 (21.7)	–
Existing risk factors-no. (%)	–	–	–
Smoking	824 (45.1)	21 (47.8)	0.72
Alcohol consumption	236 (12.9)	5 (10.9)	0.68
Hypertension	860 (47.1)	15 (32.6)	0.052
Hyperlipidemia	705 (38.6)	11 (23.9)	0.043^*^
Diabetes mellitus	342 (18.7)	6 (13.0)	0.33
Chronic kidney disease	13 (0.7)	1 (2.2)	0.30
Chronic liver disease	21 (1.2)	0 (0)	1.00
Cerebrovascular events	39 (2.1)	1 (2.2)	1.00
Chronic obstructive pulmonary disease	43 (2.4)	0 (0)	0.62
History of general anesthesia	411 (22.5)	6 (13.0)	0.13
History of cardiac infarction	320 (17.5)	7 (15.2)	0.68
ASA status-no. / total no. (%)	–	–	0.48
I	11 (0.61)	1 (2.2)	–
II	533 (29.5)	16 (34.8)	–
III	1167 (64.5)	27 (58.7)	–
IV	97 (5.4)	2 (4.3)	–
NYHA grade	–	–	0.53
Lower than Grade 1	510 (27.9)	9 (20.0)	–
Grade 1	177 (9.7)	7 (15.2)	–
Grade 2	779 (42.7)	21 (45.7)	–
Grade 3	340 (18.6)	9 (20.0)	–
Grade 4	20 (1.1)	0 (0)	–
Ejection fraction (%)	61.2 ± 8.7	62.3 ± 7.6	0.40
Duration of surgery-min	225.2 ± 82.4	195.0 ± 52.0	0.013^*^
Cardiopulmonary Bypass-no. (%)	1321 (72.3)	30 (68.2)	0.95
Duration of CPB-min	106.2 ± 47.4	91.2 ± 40.0	0.071
Aortic clamping time-min	71.7 ± 32.1	62.6 ± 33.3	0.11

Plusminus values are means ± SD

*Abbreviations*: *BMI* denotes body-mass index, *ASA* denotes American Society of Anesthesiologists, *NYHA* grade denotes New York Heart Association grade, *CPB* is cardiopulmonary bypass

**Table 4:** Logistic regression analysis of risk factors for confirmed awareness

	B	S.E.	Wald	df	*P* value	OR	95 % CI for OR	
							Lower	Upper
Hyperlipidemia	−0.504	0.358	1.984	1	0.159	0.604	0.300	1.218
Age	−0.623	0.369	2.854	1	0.091	0.536	0.260	1.105
Duration of Operation	−0.006	0.002	5.593	1	0.018	0.094	0.990	0.999
Constant	−1.650	0.623	7.006	1	0.008	0.192	–	

*Abbreviations*: *OR* denotes odds ratio, *CI* confidence interval

## Discussion

### Incidence

In the present study, we found the awareness rate in cardiac surgery was 2.5 %, which was considerably higher than that in general surgery [2, 9–12]. Several factors may have contributed to the higher occurrence of anesthesia awareness. Patients are particularly vulnerable to awareness during painful procedures such as sternotomy, electrocauterization, or any surgical manipulations and strong stimulations like endotracheal intubation [3]. Publications what address risk factors for more frequent awareness in cardiac procedures also mention compromised hemodynamics, insufficient anesthesia or analgesia that may have resulted from underdosing of anesthetic agents related to a patient’s specific requirements [13], and alterations in pharmacokinetics or pharmacodynamics of drugs during cardiopulmonary bypass [14].

The awareness rate in this investigation differed notably from other studies in cardiac surgery [2–4, 15–17]. The differences might be explained by methodology [18], human factors [18], and race; other factors that may have influenced the assessed awareness rate were the definition of awareness. Study methodology, including the number of patient interviews and especially the time elapsed after surgery when the patients were interviewed, and characteristics of the patients, as well as the number of patients evaluated. Another factor that likely influences inter-study comparability are the differences in routine practice between hospitals and between anesthesiologists, which is difficult to control in an analysis. Also, the ethnic difference may partially lead to the different results. Non-Chinese patients were enrolled in most of the prior studies, whereas all the patients in the present study were Ethnic Chinese. An ethnic difference has been reported in certain fields, like blood coagulation function and the fibrinolysis system [19, 20], along with differences in responses to special drugs, such as warfarin [21, 22].

### Distribution of the timing of intraoperative awareness

In this study, 16 patients reported events that occurred after anesthesia induction, but prior to the skin incision, of which mostly are endotracheal intubation and central line placement. Although analgesics like fentanyl will blunt hemodynamic responses to intubation to some degree, attempting to attenuate arousal of cerebral cortical activity has failed [23]. When a difficult airway is presented, multiple strong stimulations of intubation attempting might contribute to more frequent occurrences of explicit recall. Meanwhile, the depth of anesthesia will lower down once the maintenance of anesthesia is not scheduled during the attempting. During operation 18 patients experienced awareness, not being able to recognize the exact timing of the events. Only few events were well identified by specific time or manipulations, especially auditory perceptions and general manipulations that might occur throughout surgery. Five complaints occurred at the end of the surgery, which was when the depth of anesthesia was lowest to adapt to the reduced stimuli and avoid further compromising hemodynamics.

### Michigan Awareness Classification

Confirmed awareness graded by the Michigan Awareness Classification is shown in Fig. 3. While the majority of awareness events in Classes 1 and 2 were related to auditory and tactile perceptions (39 of the 46 confirmed awareness events), only seven cases with mild-to-severe pain, with or without a sensation of paralysis, were classified as Grade 3 to 5. Meanwhile, 11 patients (24 %) experienced distress from fear, nausea and being unable to speak. In a previous prospective randomized trial, five out of nine patients had confirmed awareness of Class 1 and 2, and five out of nine cases experienced distress [24]. These finding demonstrated that patients were more likely to experience a lower class of intraoperative awareness. However, distress during the period of awareness was frequently found. Therefore, careful postoperative follow-up should be arranged for patients complaining of awareness.

### Hyperlipidemia and awareness

As shown in Table 3, patients who reported confirmed awareness had a shorter duration of surgery (195.0 ± 52.0 min vs. 225.2 ± 82.4 min, *p* = 0.013, OR = 0.994, 95 % CI = 0.990–0999), compared with the other patients. However, as the odds ratio is proximal to the value 1, duration of operation won’t be considered as a significant index for establishing a risk factor for the patients’ intraoperative awareness.

Interestingly, this study indicated that hyperlipidemia was negatively related to anesthesia awareness using chi-square tests, demonstrating that hyperlipidemia is a potential protective factor against intraoperative awareness in cardiac surgery. As the elder people suffer hyperlipidemia more frequently, to clarify that hyperlipidemia is an independent protective factor rather than a coincidence with old age, a logistic regression where age, duration of operation and hyperlipidemia were entered into was done and found no association between hyperlipidemia and awareness.

However, an animal experiment conducted in mice revealed that high cholesterol level increases the anti-nociceptive effect of opioids and an analysis of the clinical records in the China-Japan Friendship Hospital (Beijing, China) was carried out to conclude that there exists a reverse correlation between the serum cholesterol and opioid efficacy in human [25]. As patients are subjected to awareness more often during painful procedures such as sternotomy and strong stimulations such as endotracheal intubation [3], the enhanced analgesia effect of opioids in the patients with hyperlipidemia like hypercholesterolemia will lower down the occurrence of anesthesia awareness. Nevertheless, a prospective cohort study is needed to reveal the relationship between hyperlipidemia and awareness. The coming era of translational medicine promises to clarify whether genetic variations contribute to a possibly lower risk of intraoperative awareness among patients with hyperlipidemia.

### Limitations

One of the limitations would be the timing for interviews. Since it is difficult to determine when patients will regain consciousness and as a result of the disability to communicate while still remain intubated for compromised hemodynamics, it is tough for the researchers to initiate the first interview at the appropriate time. Similarly, the patients would be discharged after surgery in 1 to 2 weeks, making it relative limited time for research staff to conduct the follow-up questionnaires. Since patients mostly report intraoperative awareness within 30 days, the true incidence of awareness may have been higher.

Another consideration is that the lack of appropriate hallmarks for identifying the specific timing of each reported events had complicated the analysis of the risk factors.

## Conclusions

Patients undergoing cardiac surgery are at relatively higher risk for intraoperative awareness. Distress during awareness as an outstanding discomfort during the surgery with implications for postoperative psychological complications in the hospital and long-term outcomes following the awareness, deserves the attention of anesthesiologists. Hyperlipidemia is a potential protective factor for intraoperative awareness for patients receiving cardiac surgery.
